# An *Ac/Ds*-mediated gene trap system for functional genomics in barley

**DOI:** 10.1186/1471-2164-10-55

**Published:** 2009-01-29

**Authors:** Katina Lazarow, Stephanie Lütticke

**Affiliations:** 1 Biocenter Klein Flottbek, University of Hamburg, Ohnhorststrasse 18, 22609 Hamburg, Germany; 2 Institute of Biology/Applied Genetics, Free University Berlin, Albrecht-Thaer-Weg 6, 14195 Berlin, Germany

## Abstract

**Background:**

Gene trapping is a powerful tool for gene discovery and functional genomics in both animals and plants. Upon insertion of the gene trap construct into an expressed gene, splice donor and acceptor sites facilitate the generation of transcriptional fusions between the flanking sequence and the reporter. Consequently, detection of reporter gene expression allows the identification of genes based on their expression pattern. Up to now rice is the only cereal crop for which gene trap approaches exist. In this study we describe a gene trap system in barley (*Hordeum vulgare *L.) based on the maize transposable elements *Ac/Ds*.

**Results:**

We generated gene trap barley lines by crossing *Ac *transposase expressing plants with multiple independent transformants carrying the *Ds *based gene trap construct GT*Ds*B. Upstream of the β-Glucuronidase start codon GT*Ds*B carries splice donor and acceptor sites optimized for monocotyledonous plants. DNA blot analysis revealed GT*Ds*B transposition frequencies of 11% and 26% in the F_1 _and F_2 _generation of gene trap lines and perpetuation of transposition activity in later generations. Furthermore, analysis of sequences flanking transposed GT*Ds*B elements evidenced preferential insertion into expressed regions of the barley genome. We screened leaves, nodes, immature florets, pollinated florets, immature grains and seedlings of F_2 _plants and detected GUS expression in 51% (72/141) of the plants. Thus, reporter gene expression was found in 24 of the 28 F_1 _lines tested and in progeny of all GT*Ds*B parental lines.

**Conclusion:**

Due to the frequent transposition of GT*Ds*B and the efficient expression of the GUS reporter gene, we conclude that this *Ac/Ds*-based gene trap system is an applicable approach for gene discovery in barley. The successful introduction of a gene trap construct optimized for monocots in barley contributes a novel functional genomics tool for this cereal crop.

## Background

Gene trapping has proved to be an effective strategy for functional genomics and gene discovery in both animals and plants [1-3]. Gene trap constructs are designed to detect the expression of a chromosomal gene upon insertion into its transcribed region. Consequently, the inserted gene trap reports the gene expression pattern and a visible mutant phenotype is not required for gene identification. The direct visual assessment of reporter gene expression enables the identification of functionally redundant genes, genes that operate in multiple developmental stages and genes whose functions in later development are obscured by an early lethal phenotype, all of them not easily amenable to classic genetic analysis. Several types of "trapping" systems, differing in the reporter gene constructs used, have been developed: enhancer trap, gene trap and promoter trap [2,3]. The gene traps are characterized by splice acceptor sites and sometimes an intron upstream of the reporter gene coding region. These structural features facilitate the production of in-frame reporter protein fusions regardless of insertion into intron or exon sequences.

Due to extensive knowledge about their transposition features, *Activator *(*Ac*) and *Dissociation *(*Ds*) transposable elements from maize have been successfully utilized for insertional mutagenesis in heterologous plants [4]. With the aim to discover genes whose knockout does not display a visible mutant phenotype, *Ac/Ds *based gene trap systems were introduced in *Arabidopsis *[5] and rice [6]. Furthermore, different gene trap systems based on T-DNA transfer in *Arabidopsis *[7-9] and rice [10] and on recombination in *Physcomitrella patens *[11] have proven their usefulness for the study of developmental processes and gene discovery in plants.

In addition to its agricultural importance, barley evolved as a model species for the Triticeae [12,13]. Due to gene synteny and colinearity among the Triticeae genomes [14,15] the diploid barley is considered a reference species especially for polyploid Triticeae members like wheat. Similar to maize and wheat the 4873 Mb barley genome [16] is partitioned into gene-rich regions and large stretches of gene-poor repetitive DNA composed of numerous retrotransposons [17,18]. For barley many genomics resources exist, including more than 30 well-characterized genetic linkage maps, a large-insert Bacterial Artificial Chromosome (BAC) library and a barley microarray [13,19,20]. At present, more than 400 000 expressed sequence tags (ESTs) are available [21] that cover a significant portion of the barley gene repertoire. The establishment of transformation systems [22-24] and the successful introduction of *Ac/Ds *elements [25,26] were the initial steps towards gene tagging approaches in barley [25,12,27,28].

Up to now, gene trap and enhancer trap approaches in monocots have exclusively been reported in rice [6,10,29,30]. In this study, we report the introduction of an *Ac/Ds*-based gene trap system in barley, thus expanding the number of genomics tools available to the barley research community. A gene trap construct [31] designed to provide an increased gene trapping efficiency, particularly in monocotyledonous plants, was used to produce barley gene trap lines. The frequent transposition of the gene trap construct and efficient expression of the reporter gene in these lines demonstrate that this approach is a significant step towards large-scale gene trapping in this crop.

## Results

### Generation of gene trap lines

**Figure 1:**
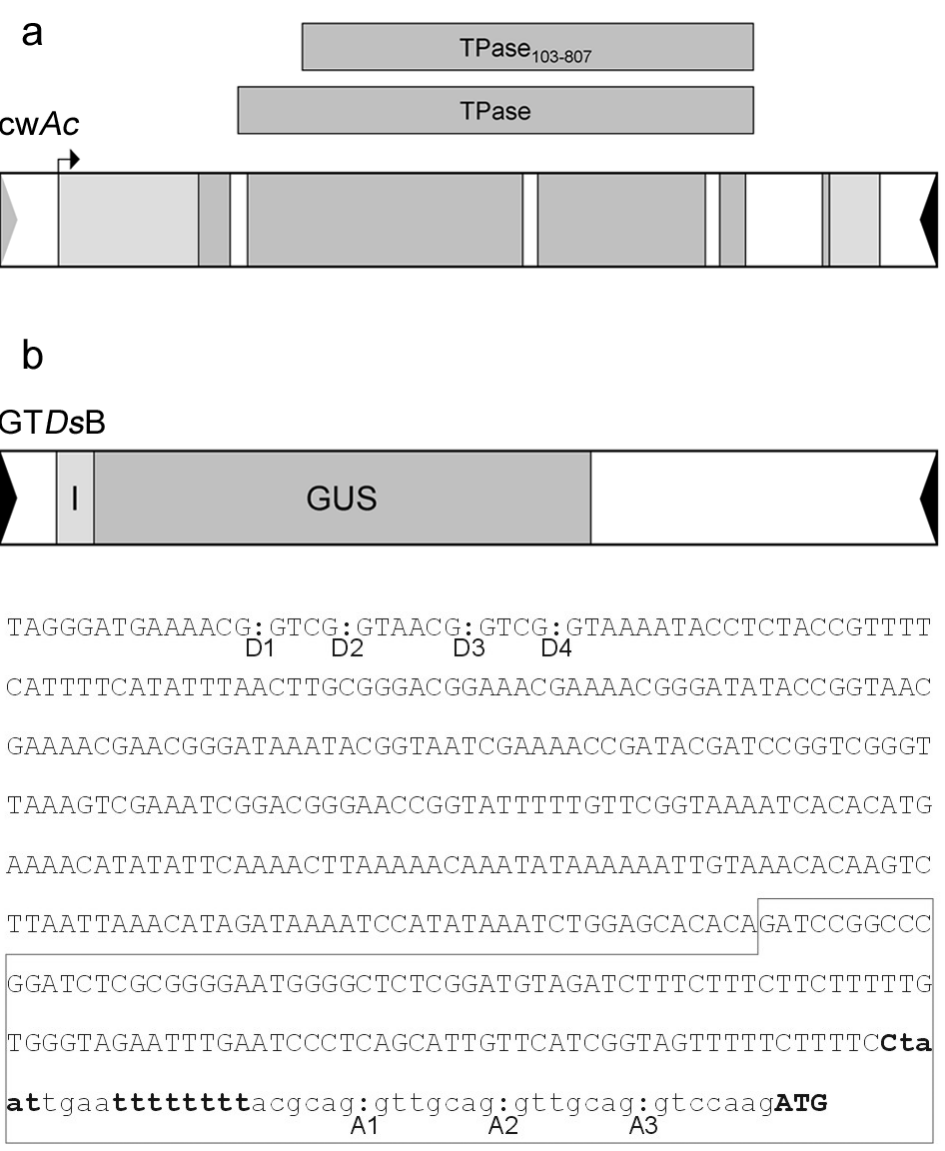
**Schematic diagrams of the *Ac/Ds*-derived constructs used for generation of gene trap lines**. **a **The two cw*Ac *constructs express a full-length 807 amino acids TPase and a N-terminally truncated 705 amino acids TPase_103–807_, respectively, under control of the native *Ac *promoter. Both elements were immobilized by disruption of the 5' terminal inverted repeat (gray arrowhead). The intact 3' terminal inverted repeat is shown as a black arrowhead. A small arrow indicates the transcription start. TPase protein coding sequences are indicated by dark-gray boxes. **b **The *Ds*-based gene trap construct GT*Ds*B contains the *uidA *coding region (GUS), the 3' sequences of the first *Act1 *intron from rice and a synthetic triple splice acceptor site (I).*Ac *sequences are depicted as an open box and the terminal inverted repeats are shown as filled arrowheads at the outer ends. The sequences of the putative splice donors (D1-D4), the synthetic splice acceptor sites (A1-A3) and the *Act1 *intron (boxed sequence) existing in the GT*Ds*B 5' subterminal region are shown. The branchpoint sequences and T-tract are bold typed.

The maize transposable element *Ac/Ds *was chosen to construct a two-component gene trap system for barley. Two versions of *Ac *expressing either wild type transposase (TPase) or an N-terminally truncated transposase (TPase_103–807 _[32]) under control of the native *Ac *promoter were used (Figure 1a). Both TPase-expressing elements were immobilized by removal of the five terminal bases from the 5' terminal inverted repeat (TIR) sufficient to abolish *Ac *transposition [33]. The non-autonomous *Ds *element named GT*Ds*B carries the *uidA *reporter gene encoding β-glucuronidase (GUS) [31]. The reporter gene is preceded by engineered intron and triple splice acceptor sequences upstream of the ATG codon (Figure 1b). Each of the three constructs was stably transformed into barley cultivar Golden Promise by particle bombardment. To verify the integration of intact copies and estimate the transgene copy number, in order to select parental lines for crosses, we subjected independent lines, seven carrying *Ac *and 34 harbouring GT*Ds*B, to DNA gel blot analysis. Eleven GT*Ds*B lines with low (one to three), medium (four to seven) and high (up to 12) copy number and four TPase lines were selected as starter lines (Table 1). Two TPase lines express wild type TPase and two the truncated TPase_103–807 _protein. The number of integrated *Ac *TPase copies was between one and four in the different lines. The expression of a functional TPase was confirmed with plants from all four TPase lines (C.K. Friedrich, personal communication) using a transient assay for TPase activity [34]. From crosses of the four TPase lines with each of the 11 GT*Ds*B lines we obtained F_1 _progeny for 30 different combinations.

**Table 1:** GT*Ds*B transposition in F_1 _and F_2 _generation of gene trap lines

Parental lines			F_1 _generation		F_2 _generation				
		
GT*Ds*B line	GT*Ds*B copies	TPase line^a^	No. plants analysed	No. with tnp^b^	F_2 _parent	No. plants analysed	No. with tnp^b^	Independent tnp^c^	No. pa^d^
2a/d	1–2	2	3	-	GT32	6	3	1	-
		4	9	-	GT73	4	4	1	-
		7	1	-	-				
23	1–2	2	4	-	GT33	4	1	1	-
		3	2	1	GT29	4	4	3	-
		4	1	-	GT39	1	1	1	1
		7	4	3	GT41	5	5	2	-
31B	2	4	4	-	GT49	4	2	1	1
		7	5	2	GT54	5	3	1	-
6a/c	2–4	2	1	-	GT70	4	-	-	-
		4	1	-	GT67	7	-	-	1
		7	2	-	GT4	7	5	5	1
31A	4	2	2	-	GT38	6	6	2	-
		4	1	-	-				
		7	6	1	GT52	5	-	-	-
16	3–5	2	2	-	GT19	4	3	1	-
		7	2	-	GT37	7	2	2	-
		7	2	-	GT40	4	4	2	-
14A	5–6	4	2	-	GT13	4	3	1	1
		7	3	-	GT22	5	-	-	2
11	5–8	3	1	1	GT82	3	3	1	-
		4	1	-	GT66	3	-	-	1
		7	2	-	GT80	54	16	10	3
10	6–7	2	4	-	GT9	7	-	-	-
		4	3	-	GT2	7	-	-	5
		7	2	-	GT6	7	1	1	-
14B	8–10	2	2	-	GT23	4	2	2	-
		7	3	1	GT15	8	3	3	1
26	12	2	2	-	GT35	4	3	3	1
		7	2	-	GT63	8	5	5	3

Total			79	9		191	79	49	21

^a^TPase line 3 and 4 carry four and two copies of the TPase construct respectively, whereas 2 and 7 carry one and four TPase_103–807 _constructs respectively

^b^Number of plants showing GT*Ds*B transposition (tnp)

^c^Number of plants with independent transposition events

^d^Number of plants with phenotypic abnormality (pa)

### Analysis of GT*Ds*B transposition

DNA gel blot analysis was employed to study the transposition of GT*Ds*B in the gene trap lines. In these experiments the occurrence of a new GT*Ds*B-hybridizing DNA fragment in comparison to the corresponding GT*Ds*B parental line was chosen as a criterion to indicate transposition of GT*Ds*B. We performed analysis of GT*Ds*B excision and reinsertion events in 79 F_1 _plants originating from 29 independent crosses. Nine plants (11%) derived from six independent crosses showed novel hybridizing bands that were not present in the parental GT*Ds*B lines (Table 1).

**Figure 2:**
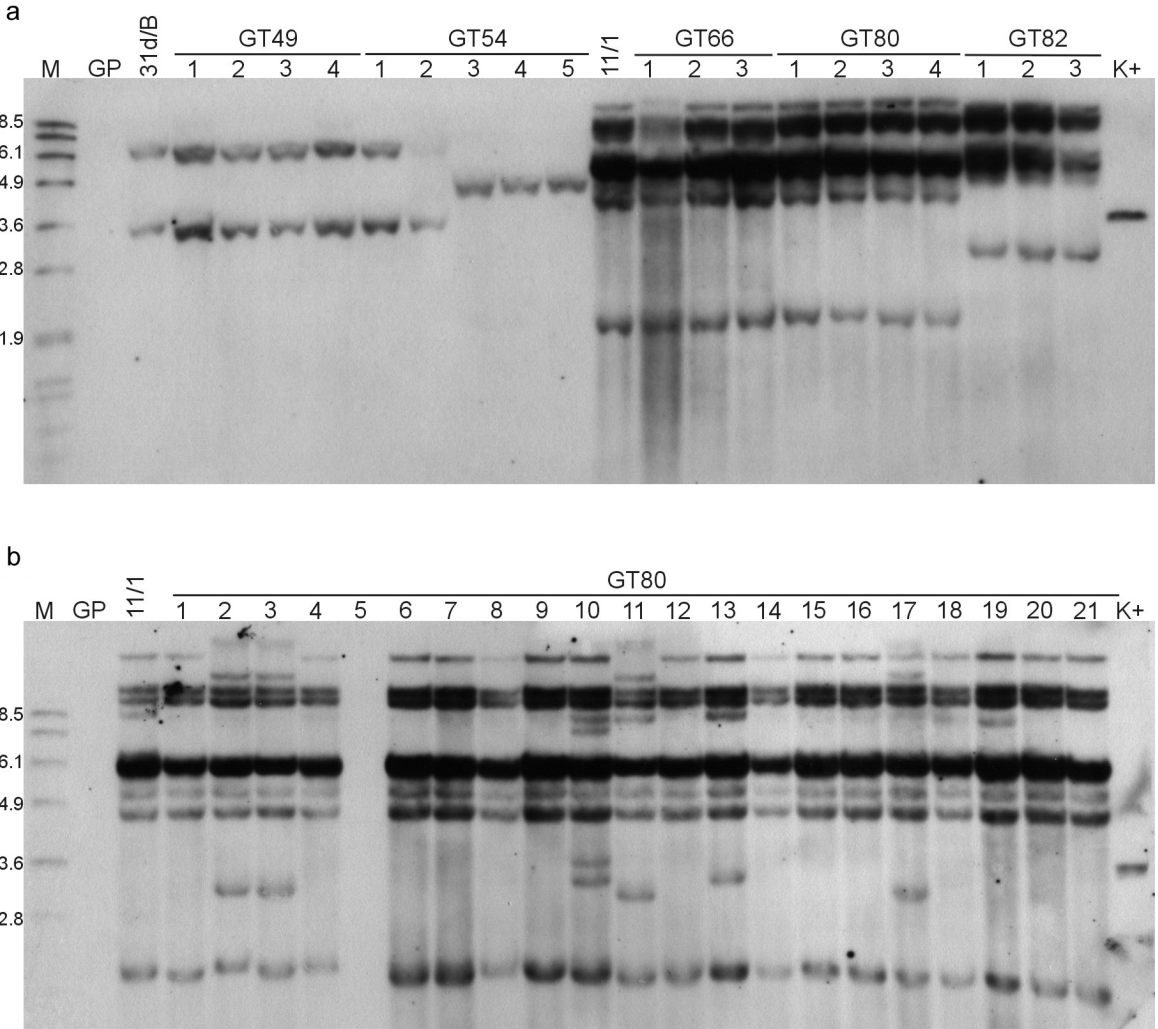
**Representative DNA gel blot analysis of F_2 _plants derived from five independent crosses**. Genomic DNA of three to five F_2 _siblings from lines GT49, GT54, GT66, GT80 and GT82 (a) and of 21 F_2 _siblings from line GT80 (b), as well as DNA of the GT*Ds*B parental lines (31d/B and 11/1) and Golden Promise wild type (GP) were digested with *Bgl*II and blots were hybridized to a GUS probe. The numbers above each panel announce the individual plant identification, e.g. GT49/1 in lane 4 (a). The numbers on the left side of each panel indicate the positions of size markers (M) in kb.

For a second set of experiments we rescued progeny harbouring both TPase and GT*Ds*B constructs from 28 selfed F_1 _plants (F_2 _parent, Table 1), each derived from an independent cross of different parental lines. A total of 191 F_2 _plants, including an average of five siblings per independent cross, with the exception of lines GT39 and GT80 with one and 54 plants each, were analyzed. New GT*Ds*B-hybridizing bands were detected in 79 F_2 _plants (41%) representing 21 of the 28 F_1 _gene trap lines (75%). Examples of DNA hybridization patterns are shown in Figure 2. Unique hybridization patterns, suggesting independent transposition events, were found in 49 F_2 _plants (26%). Independent transposition events can be due to either transposition in independent cells of the F_1 _plant, which subsequently were transmitted to progeny, or to somatic transposition in the F_2 _seedling (for example see Figure 2b, plants GT80/10 and GT80/13). In contrast, early transposition of GT*Ds*B in the F_1 _generation may result in all progeny inheriting the same insertion (for example see Figure 2a, GT82/1–3). Additionally a transposition event having occurred in one of the barley tillers during the development of the F_1 _plant may lead to several but not all F_2 _siblings showing the same new GT*Ds*B insertion (for example see Figure 2a, GT54/3–5). Transposition of GT*Ds*B occurred in progeny of crosses with each of the 11 GT*Ds*B parental lines (Table 1), evidencing that every independent parental GT*Ds*B line carries transposition competent constructs.

The gene trap F_2 _population was also screened for visible phenotypic abnormalities. In 21 of the 191 (11%) gene trap F_2 _plants deviations from barley wild type phenotype were observed (Table 1). These included reduced fertility (4/21), aberrant leaf pigmentation (4/21) and stunted growth (9/21) (data not shown). Interestingly, in two plants, GT29/6 and GT39/6, showing asymmetric internodes leading to bending stems and stunted growth with shortened ears respectively, the phenotypic deviation coincided with an independent transposition event. A possible connection between the transposition event and the conspicuous phenotype must be examined in further experiments.

To prove the persistence of GT*Ds*B transpositional activity in later generations, we subjected a total of 252 F_4 _plants originating from the F_2 _plants GT54/8, GT29/6, GT49/7 and GT41/3 to DNA gel blot analysis. In five of the 67 GT54/8 progeny (7.5%), 22 of the 64 GT29/6 progeny (34.4%), 10 of the 59 GT49/7 progeny (17%) and three of the 64 GT41/3 progeny (4.8%) new GT*Ds*B-hybridizing DNA fragments were detected. These transposition events must have occurred either in the F_2 _and F_3 _generation or in somatic tissue of the F_4 _plants.

### Sequence analysis of GT*Ds*B flanking regions

We employed TAIL-PCR [35] to obtain DNA sequences flanking transposed GT*Ds*B constructs from gene trap F_2 _plants. In total, 32 genomic sequences ranging from 111 to 678 bp were isolated and compared to publicly available databases using BLAST searches. We considered Expectation (E) values below 1e-6 to assign a putative identity to a flanking sequence. As evidenced by similarity to expressed sequence tags (ESTs) from members of the Triticeae and maize, 19 of the 32 GT*Ds*B insertions (59%) are located in transcribed genomic regions (Table 2). Moreover, 15 GT*Ds*B flanking sequences (47%) show high identity to ESTs from barley (Table 2). Assuming an average sequence length of about 400 bp, the 419 146 non-overlapping barley ESTs in the databases [21] represent approximately 168 Mb of total sequence, or about 3% of the 4873 Mb barley genome [16]. Consequently, a random fragment of barley DNA would have on average a 3% chance of being homologous to a barley EST in the database. Considering that more than 80% of the barley genomic sequences are intergenic heterochromatin [36] and therefore not expressed, the frequent identity of the flanking genomic sequences to barley ESTs clearly indicates a preference for GT*Ds*B insertion into coding regions.

**Table 2:** GT*Ds*B flanking sequences with similarity to ESTs in public databases

F_2 _plant	Length (bp)	Organism	Accession number	E-value^a^	Similarity (%)
GT4/7	603	*H. vulgare*	CB881303.1	6e-79	91
	517	*H. vulgare*	CB880126	2e-97	89
GT29/1	561	*H. vulgare*	CK566326	1e-09	89
	537	*H. vulgare*	U43284CDS	9e-29	88
GT37/1	111	*H. vulgare*	CA011566	1e-20	95
	378	*H. vulgare*	CB864149	6e-26	98
GT41/1	678	*H. vulgare*	BF620234	e-102	89
	376	*H. vulgare*	CB867749	9e-22	94
GT41/9	304	*T. aestivum*	CN010744	1e-66	88
	503	*H. vulgare*	BF616808	6e-36	82
	327	*H. vulgare*	AJ461421	1e-60	100
GT54/6	316	*H. vulgare*	CD056550	e-149	97
	527	*T. aestivum*	CV758685	3e-16	88
GT63/5	470	*H. vulgare*	CB864987	e-108	93
	133	*H. vulgare*	CB881931	4e-58	97
	124	*H. vulgare*	BU966953	1e-11	94
	193	*Z. mays*	DN560626	9e-83	95
GT82/4	292	*H. vulgare*	AJ473098	2e-77	98
	356	*T. aestivum*	BQ170291	2e-13	88

^a^Statistical significance threshold for reporting matches against database sequences obtained after BLAST program search

### Expression of the GUS reporter

The expression of the GUS reporter gene was assayed by histochemical GUS staining in 141 F_2 _plants, comprising 1 to 8 progeny of the 28 individual F_1 _gene trap lines (Table 3). Staining for GUS expression was performed in leaves, nodes, immature florets, pollinated florets, immature grains and seedlings covering several stages of barley development. The leaves, nodes and immature florets were collected from developmental stage 49 defined following the Zadoks code system for growth staging in barley [37]. Pollinated florets, immature grains and seedlings represent developmental stages 65–69, 83–85 and 10 respectively. For the majority of the 141 F_2 _plants multiple explants were examined (one leaf, two nodes, 8 pollinated florets, 20 immature florets, 8 immature grains and 8 seedlings), resulting in a total of 5134 analysed explants. This experimental approach was chosen to enable the distinction of somatic from heritable events. We assume that, if only one explant of a certain organ type shows GUS expression, it may be considered as a somatic event. In contrast, if the majority or all explants of the same organ type from a single plant exhibit an equal GUS expression pattern, the event may be transmitted to progeny. Due to the two-element approach, these inheritable events can be stabilized by segregation of the TPase construct and are amenable to further analysis.

**Table 3:** GUS expression in the F_2 _generation of gene trap lines

GT*Ds*B line	Gene trap line	No. plants analysed	No. plants with GUS	Organ with GUS activity (no. explants)
2a/d	GT32	6	2	grain (1), seedling (1)
	GT73	4	1	grain (1)
23	GT33	4	-	-
	GT29	4^(1c)^	2	pollinated floret (1), seedling (1)
	GT39	1	-	-
	GT41	5	3	pollinated floret (13), grain (4), seedling (1)
31B	GT49	4	2	pollinated floret (2), seedling (1)
	GT54	5	5	immature (7)/pollinated (1) floret, grain (10), seedling (15)
6a/c	GT70	4	1	pollinated floret (1), node (1)
	GT67	6	4	node (4), leaf (1)
	GT4	7^(1a)^	-	-
31A	GT38	6	1	seedling (1)
	GT52	5^(1d/1e)^	4	node (1), grain (10)
16	GT19	4^b^	1	grain (1)
	GT37	8	2	grain (4), seedling (1)
	GT40	4	-	-
14A	GT13	4^(2b/2c)^	2	grain (1), seedling (7)
	GT22	5^(4b/1c)^	4	grain (2), seedling (17)
11	GT82	3	3	immature floret (1), node (3), leaf (1)
	GT66	3^(*f*)^	2	pollinated floret (3), grain (1)
	GT80	4	4	immature floret (3), pollinated floret (2), node (4), leaf (1), grain (9), seedling (2)
10	GT9	7^(7b)^	2	grain (2), seedling (2)
	GT2	7^(6b/1c)^	5	grain (2), seedling (4)
	GT6	7^(5b/2c)^	4	grain (6), seedling (3)
14B	GT23	4^(2a/1d)^	1	seedling (1)
	GT15	8^(1b/3c)^	7	pollinated floret (9), grain (12), seedling (17)
26	GT35	4	4	immature floret (41), pollinated floret (32)
	GT63	8	7	immature floret (3), grain (23), seedling (2)

Total		141	72	

^(No. plants a)^Only seedlings were analysed

^(No. plants b)^Only grains and seedlings were analysed

^(No. plants c)^Only pollinated florets, grains and seedlings were analysed

^(No. plants d)^No pollinated florets were analysed

^(No. plants e)^No grains were analysed

^(No. plants f)^Only immature florets, nodes and leafs were analysed

**Figure 3:**
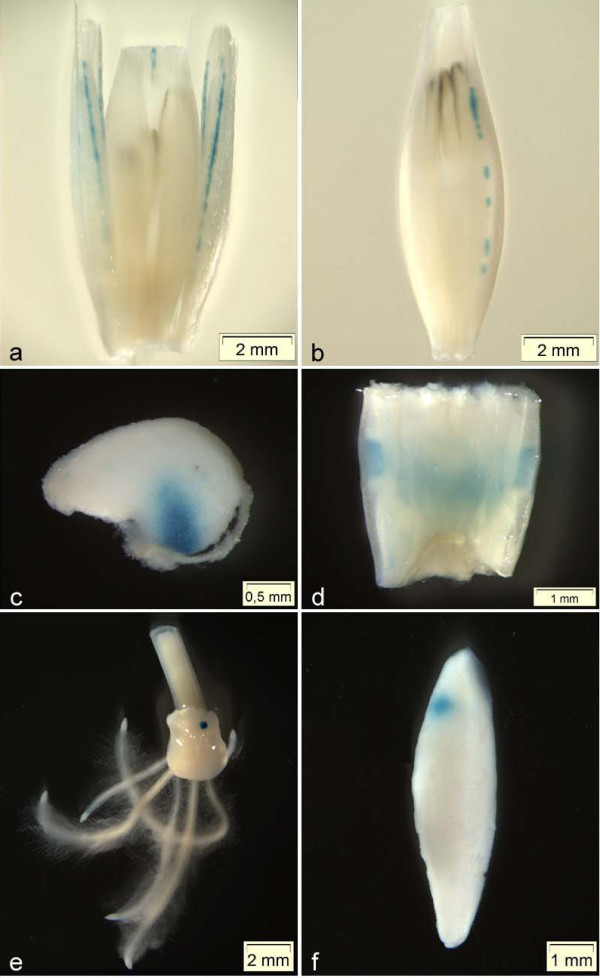
**Examples of GUS expression in F_2 _plants**. **a **GUS staining in the lemma of the central floret and the sterile lateral florets (GT35/8). **b **GUS staining in the lemma (GT29/6). **c **GUS staining in endosperm tissue (GT15/9). **d **GUS staining in the node (GT4/10). **e **GUS staining in scutellar tissue of a seedling (GT37/6). **f **GUS staining in endosperm tissue (GT80/1).

Expression of the GUS reporter could be detected in 51% (72/141) of the analysed F_2 _plants (Table 3). Moreover, GUS expression was found in 24 of the 28 F_1 _lines and in progeny of all GT*Ds*B parental lines used. Examples of GUS expression in various organs are shown in Figure 3. In immature florets and in pollinated florets GUS activity was primarily detected in the palea and lemma (for examples see Figure 3a and 3b). In addition, in three cases GUS activity appeared in the stigma and pistil (data not shown). In all samples the GUS signals in culm nodes corresponded to the example shown in Figure 3d. The seedlings displayed GUS expression primarily in the scutellum (for example see Figure 3e). In five cases GUS activity could be observed in the roots (data not shown). In the majority of GUS positive seeds the expression was localized in the endosperm (for example see Figure 3c and 3f). However, in three cases GUS signals were observed in the pericarp (data not shown).

Out of the 72 GUS positive plants 45 showed GUS expression restricted to one or two explants, even when occurring in several organs, indicating that the majority of the events is due to somatic transposition of GT*Ds*B. In such cases only a limited portion of somatic tissue carries the new GT*Ds*B insertion and can consequently be expected to express GUS. In contrast, in 14 F_2 _plants GUS expression was detected in the same tissue in at least 50% of the explants (Table 4) denoting candidates for heritable events. In eight of these candidates GUS expression was detected in scutellar tissue of seedlings (GT54/5-GT15/4) or in the endosperm of immature grains (GT63/4). Analysis of progeny will confirm the heritability of these gene trap events enabling identification of GT*Ds*B integration sites.

**Table 4:** Gene trap F_2 _plants with GUS expression in at least 50% of the explants

F_2 _plant	No. of explants analysed	No. of explants with GUS	Explant type
GT41/3	16	13	pollinated florets
GT54/5	8	5	seedlings
GT54/10	8	5	seedlings
GT13/8	8	5	seedlings
GT22/3	8	6	seedlings
GT22/5	8	8	seedlings
GT15/2	8	5	seedlings
GT15/4	8	4	seedlings
GT15/9	9	9	pollinated florets
GT35/1	8	8	pollinated florets
GT35/3	8	8	pollinated florets
GT35/7	8	8	pollinated florets
GT35/8	8	8	pollinated florets
GT63/4	16	9	grains

The GUS staining frequency ranged between 3% and 26% in individual organs (Table 5). As expected, the highest frequencies of 26% and 24% were observed in grains and seedlings representing mostly tissues of the F_3 _generation. As a consequence of transposition in F_3 _tissues early events of the F_3 _generation can be detected in addition to events that occurred in the preceding generations.

**Table 5:** GUS expression frequency in various organs of F_2 _plants

		GUS positive plants	
		
Organ	No. of F_2 _plants tested	No.	%
leaf	99	3	3
node	99	13	13
immature floret	99	9	9
pollinated floret	104	14	13
grain	134	35	26
seedling	138	33	24

### Analysis of spliced GUS transcripts

The expression of GUS depends on the transcriptional fusion between the reporter open reading frame and upstream gene sequences following the insertion of GT*Ds*B into a transcription unit. Consequently, correct and efficient splicing of the gene trap construct by the host spliceosome is crucial and has been already shown for GT*Ds*B in transient experiments [31]. We aimed to demonstrate that splicing of stably integrated GT*Ds*B constructs in the gene trap barley lines is accomplished just as accurately. For these experiments the gene trap line GT35 was chosen, since the same GUS expression pattern (Figure 3a) was found in 100% of the pollinated florets in all progeny tested, indicating an inheritable gene trap event (Table 4). Additionally, RNA gel blot analysis confirmed the occurrence of *uidA*-specific transcripts exceeding the size of the original *uidA *transcript by 1.1 and 0.4 kb, thus indicating the presence of transcriptional fusions encoding GUS in the florets of gene trap line GT35 (data not shown). We used 5'-RACE (rapid amplification of cDNA ends [38]), to isolate spliced transcripts encoding GUS. Out of 17 isolated 5' sequences, in 14 the splice site A1 and in three A2 has been properly used to generate the reporter gene transcript. These findings are consistent with previous studies of GT*Ds*B splice products in transiently transformed barley tissue, revealing that the splice acceptor sites A2 and A3 were utilized with almost equal frequencies but eleven times less frequent than A1 [31].

## Discussion

With the development of an *Ac*/*Ds *based gene trap system in barley we contribute a novel functional genomics tool for this species. In our approach gene trapping efficiency depends on transposition of the GT*Ds*B construct. DNA gel blot analyses indicate frequent transposition of the GT*Ds*B element in the gene trap lines. The transposition frequency of 11% (9/79) detected in the F_1 _generation is in the range of the transposition frequency presented for the barley activation tagging system [28], but higher than that reported for transposition of *Ds *elements (2%) and autonomous *Ac *elements (1.5%) in F_1 _and T_1 _generations of barley [25,26]. In the F_2 _generation we observed in 26% (49/191) of the plants unique newly transposed GT*Ds*B elements, indicating a transposition frequency sufficient for large-scale mutagenesis screens in barley [28]. In addition, the rapid recovery of many independent GT*Ds*B insertions will be potentiated by independent transposition events in the tillers of a single barley plant.

In rice and *Arabidopsis *extensive collections of insertion lines have been generated by high throughput T-DNA transformation. However, for large-genome and transformation-recalcitrant species like barley insertion mutagenesis strategies based on transposable elements are likely to be advantageous. A recent detailed study of T-DNA insertion distribution in rice revealed a preference for insertion into genic sequences, thus reducing the number of insertions needed to saturate the genome [39]. The barley genome supposedly contains the same number of genes like rice, but is due to amplification of gene-poor regions about 12 times larger [17]. Therefore, insertion mutagenesis merely based on T-DNA transformation would require far more than 200 000 primary transformants. For barley these will be difficult to obtain given that barley transformation requires extensive tissue culture periods and is still laborious and relatively inefficient. By contrast, the transposon based approach enables with only a few initial starter lines the successive accumulation of novel independent insertions in a definite plant population [28,40]. In addition, a direct comparison of *Ac *and T-DNA insertions in aspen revealed for the transposable element a two fold higher frequency of landing into coding regions [41]. The preferential insertion into expressed genomic sequences is a feature of *Ac/Ds *transposition, that has been well documented in barley [12,13,27], *Arabidopsis *[42,43] and rice [44,45,40]. This preference we also observed in the barley gene trap lines evident in the frequent identity of transposed GT*Ds*B flanking genomic sequences to barley ESTs.

By using 11 independent GT*Ds*B starter lines with a variable GT*Ds*B copy number we generated a barley gene trap population comprising more than 40 putative GT*Ds*B launch pads at different genomic positions. The transposition of GT*Ds*B could be detected in progeny of crosses with each of the 11 GT*Ds*B parental lines, demonstrating that every independent GT*Ds*B line carries transposition competent constructs and can be utilized for gene trapping in barley. Although, currently no mapping populations are available for the barley variety used here, a sequence based strategy for assigning *Ds *insertions in Golden Promise to linkage map coordinates on the existing Oregon Wolfe Barley map has been reported [13]. Most agronomically important traits, such as yield and quality parameters, are controlled by many genes arranged as so called "quantitative trait loci" (QTLs) [46]. GT*Ds*B insertions nearby known QTLs will therefore provide useful launch pads for local saturation mutagenesis [47]. This approach will take advantage of the well documented *Ac/Ds *feature of preferential transposition to chromosomally linked positions [48], equally observed in barley [12,25].

Interestingly, the transposition frequency in the F_2 _generation ranges between 5% (GT*Ds*B 10) and 67% (GT*Ds*B 26), if calculated per independent GT*Ds*B parental line. The variance of transposition frequency has been frequently observed in independent barley and rice transgenic lines [25,28,40,44,45] and likewise in dicots [48]. Earlier studies have shown that *Ds *transposition is influenced by the genomic position of the element [40,49]. Our findings confirm that the transposition frequency is rather influenced by the GT*Ds*B integration site than by the number of GT*Ds*B loci. For example, unique transpositional activity of 67% and 50% was detected in gene trap lines originating from GT*Ds*B parental lines 26 and 23 having 12 and 1–2 loci respectively. The DNA gel blot results indicate some correlation between GT*Ds*B transpositional activity and TPase-expressing construct. F_2 _plants carrying one (TPase line 2) or four (TPase line 7) TPase_103–807 _copies exhibit a noticeable higher transpositional activity of 26% (10 of 39 plants with transposition) and 27% (31 of 115 plants with transposition) than F_2 _plants having two copies of wild type TPase (13%, 4 of 30 plants with transposition). This finding is consistent with earlier reports, showing that the TPase_103–807 _induces higher excision frequencies of *Ds *than wild type TPase in *Petunia *protoplasts [50,51] and transgenic tobacco [32].

Accurate and efficient splicing of the gene trap construct is a prerequisite for reporter gene expression and therefore crucial for gene trapping efficiency. GT*Ds*B was optimized for efficient splicing in monocotyledonous plants by adapting the splice acceptor sites to the monocot consensus and the introduction of synthetic branch point and T-tract sequences [31]. An important feature in the optimization process was to attenuate the first splice acceptor site (A1; Figure 1b), since for splice acceptor site selection a scanning mechanism is postulated [52]. The isolated 5' sequences of GUS fusion transcripts indicate a preference for usage of the first splice acceptor site A1 according to former findings [31]. However, the isolation of three A2-spliced GUS transcripts out of 17 analyzed indicates a decrease in A1 selection compared to the earlier transient studies [31]. With enhanced usage of A2 the chance of receiving functional GUS at a single integration site is increased by one third compared to exclusive usage of A1 emphasizing the potential of GT*Ds*B for gene trapping.

We were primarily interested in detecting reporter gene expression in the gene trap population and, opposite to other transposon-based gene trap approaches [5,6], did not select for plants with transposed GT*Ds*B constructs prior to the analysis of GUS activity. We therefore expected (i) the GUS expression frequency to be lower than the frequencies of 26% and 16% reported in *Arabidopsis *and rice respectively [5,6] and (ii) to detect a high proportion of somatic events. To raise the probability of GUS detection, we decided to screen several organs containing differentiating and dividing cells. Additionally, for the majority of the organs multiple explants per single plant were tested for GUS activity, thus enabling to discriminate between somatic and transmissible events. This screening mode accounts for the high number of gene trap insertion events detected, since GUS expression was found in 72 of the 141 F_2 _plants (51%). GUS expression was detected in progeny of all 11 GT*Ds*B lines, which is not surprising as transposition of GT*Ds*B was likewise found in progeny of crosses with all GT*Ds*B parental lines. In 14 plants (10%) GUS expression was detected in at least half of the explants of a single organ type, primarily in seedlings and florets, suggesting candidates for transmissible events and thus for gene identification. In a rice insertional mutagenesis approach employing a *Ds *based gene trap, GUS expression was observed in 8.1% of the lines analyzed [53]. However, the heritability of the events was not addressed. The higher GUS expression frequency of 26% in grains and 24% in seedlings in comparison to the remaining organs indicates the accumulation of insertion events in later generations and demonstrates the dependence of GUS expression on GT*Ds*B transposition. In F_3 _tissues, in addition to events of the preceding generations, developmentally early transposition events can lead to detectable GUS expression. By contrast, in a T-DNA based gene trap system in rice homogeneous GUS expression frequencies in leaves, roots, florets and immature grains were detected [10].

A particular challenge for plant functional genomics is the abundance of functional gene redundancy and multigene families [54,55]. About 15% of the identified genes in sequenced plant genomes are considered to be members of tandem-arrayed gene families [55]. Therefore in *Arabidopsis *less than 2% of gene knockouts are expected to display significant mutant phenotypes [54,56]. The gene trap approach may prove to be highly beneficial as indicated by the 6 to 30 times more frequent detection of GUS reporter gene expression compared to visible mutant phenotypes in *Ac/Ds*-mediated *Arabidopsis *gene trap lines [5,57]. The identification of genes whose recovery by loss-of-function mutagenesis would have been impeded either by gene redundancy or a lethal phenotype [58-61] further emphasizes that gene trap insertional mutagenesis provides a powerful genomics strategy.

## Conclusion

Barley has one of the largest genomes of all economically important cereal crops and even though more and more genomic sequence data are available various functional genomics resources will be needed to address questions of yield and stress resistance. With the *Ac/Ds*-mediated gene trap system in barley we adopt a novel functional genomics tool for this species. This will be valuable for both gene trapping and knockout mutation as well as forward and reverse genetic screens. In the gene trap lines we observed frequent transposition of the gene trap construct GT*Ds*B from multiple launch sites sufficient for large-scale mutagenesis. The recovery of individual insertion events will be further assisted by the high number of independent insertions and the preferential transposition into expressed genomic sequences. Maintenance of transposition activity over several generations makes the gene trap lines applicable for the accumulation of independent insertions in a barley gene trap plant population. The frequent detection of GUS reporter gene expression in the gene trap lines and the proper splicing of our optimized gene trap construct finally prove gene trap insertional mutagenesis to be now attainable for barley functional genomics.

## Methods

### Construct design

To generate cw*Ac *(clipped wing *Ac*) containing an immobilized *Ac *element expressing wild type TPase under the regulatory control of the native *Ac *promoter, five base pairs of the 5' *Ac *end in pJAc [62] have been deleted according to Healy et al. [33]. For cw*AcΔ102*, expressing a transposase shortened by 102 amino acids at the N-terminus (TPase_103–807_, [32]), the *Ac *element was immobilized in the same manner. The *Ds *based gene trap construct GT*Ds*B carries a promoterless *uidA *gene preceded by a triple splice acceptor site [31].

### Plant material and transformation

Immature embryos of barley (*Hordeum vulgare *L.; cv. Golden Promise) were transformed via particle bombardment either with GT*Ds*B, cw*Ac *or cw*AcΔ102*. To facilitate the selection of transgenic plants a synthetic *pat *gene (Peter Eckes, unpublished) encoding a phosphinotricin acetyltransferase that confers resistance to glufosinate-type herbicides was co-transformed. Transformation and regeneration of transgenic barley plants was performed as described by Scholz et al. [26]. Barley plants were grown in the greenhouse at 16–18°C day/12–13°C night temperatures with a 16 h photoperiod.

### Generation of the gene trap population

To generate the gene trap lines, plants carrying GT*Ds*B constructs were crossed with plants expressing either wild type transposase (TPase) or an N-terminal truncated transposase (TPase_103–807_). F_1 _plants containing TPase as well as GT*Ds*B constructs were selected by PCR. Individual F_1 _plants, also referred to as gene trap lines, were named GT followed by a unique number. For the F_2 _generation, progeny of 28 selfed F_1 _plants containing both constructs were selected in the same way. Likewise, the F_3 _and F_4 _generation was produced by two further rounds of selfing and PCR selection.

### Genomic DNA isolation and DNA gel blot analysis

Genomic DNA was extracted from several leaf tips per plant as described by Palotta et al. [63]. To determine the copy number of GT*Ds*B and TPase constructs, DNA from GT*Ds*B and TPase plants was digested with *Bam*HI, *Mun*I or *Xba*I. The integration of intact GT*Ds*B elements was proved by digestion with *Eco*RI and *Hin*dIII, which release a 3.5 kb fragment from the pGT*Ds*B transformation vector containing the complete GT*Ds*B cassette. The integration of intact TPase constructs was confirmed by digestion with *Bam*HI and *Pac*I. For analysis of transposition events the genomic DNA of F_1_, F_2 _and F_4 _gene trap plants was digested with *Bgl*II, *Bcu*I or *Bam*HI, all cutting only once in the GT*Ds*B element. Digested DNA was subjected to DNA gel blot analysis as described by Scholz et al. [26]. For the detection of GT*Ds*B elements a 637 bp *uidA *fragment was digoxigenin labeled by PCR. The TPase specific probe was prepared by labelling a 734 bp *Ac *fragment with digoxigenin.

### Isolation and analysis of GT*Ds*B flanking sequences

DNA regions flanking GT*Ds*B inserts in gene trap lines were amplified by TAIL-PCR as described [35,64]. 10 ng of genomic DNA were used as template DNA. The specific primers for the GT*Ds*B 5' end were: R-GUS-A (5'-CCCACAGGCCGTCGAGTTT-3'), R-GUS-1 (5'-CACGGGTTGGGGTTTCTACA-3') and 3pAH2-2.1 (5'-CCGTATTTATCCCGTTCGTTTTCGTTA-3'). The following arbitrary primers were used: AD1 (5'-NGTCGASWGANAWGAA-3'), AD2 (5'-GTNCGASWCANAWGTT-3'), AD3 (5'-WGTGNAGWANCANAGA-3'), AD4 (5'-NTCGASTWTSGWGTT-3'); AD5 (5'-NGTASASWGTNAWCAA-3'), AD6 (5'-TGWGNAGWANCASAGA-3'), AD7 (5'-AGWGNAGWANCAWAGG-3'), AD8 (5'-CAWCGICNGAIASGAA-3') and AD9 (5'-TCSTICGNACITWGGA-3'). Specific tertiary PCR fragments were purified from agarose gels with Recochips (TaKaRa, Shiga, Japan) and sequenced (DNA-Cloning-Service, Hamburg, Germany). The BLAST algorithm [65] was used to compare each sequence to the publicly available databases GenBank, EMBL/EBI and DDBJ.

### GUS histochemical analysis

Plant material for GUS staining was collected from gene trap F_2 _plants considering the Zadoks code system for growth staging in barley [37]. Two nodes, one leaf and 20 florets were collected from one tiller at growth stage 49. Nodes were divided, leafs dissected and florets cut from the spike. To obtain the pollinated florets eight spikelets were collected from one ear at growth stage 65–69. The awns of all florets were cut off, while sterile lateral spikelets were partially kept. At growth stage 83–85, eight grains were collected from one ear and divided after the removal of the lemma and palea. For seedling analysis, eight embryos were isolated from one ear and germinated for five days on sterile plates. Expression of GUS in barley tissues was assayed essentially as described by Jefferson [66] and Dai et al. [67]. Materials, with the exception of grains, were pre-treated with 90% acetone for 2 h at 20°C. Grains were pre-treated with 70% ethanol for 2 min. Explants were washed twice in 50 mM sodium phosphate (pH 7) transferred into microtiter wells containing GUS staining solution (50 mM sodium phosphate pH 7, 10 mM EDTA, 0,05% Triton-X 100, 100 μg/ml Chloramphenicol, 1 mM X-Gluc), vacuum infiltrated for 2 min and incubated at 37°C for 24 h. Tissues were cleared by several changes of 96% ethanol and stored in 75% ethanol. The samples were observed under a light microscope (SZX9, Olympus, Japan) and images captured using a CCD camera (ColorView, Olympus, Japan) and appropriate software (analySIS, Soft Imaging System GmbH, Münster, Germany).

### 5'RACE (rapid amplification of cDNA ends)

Total RNA was extracted as described by Chomczynski and Sacchi [68] from sterile lateral spikelets of gene trap line GT35. 1 μg was used as a template in a reverse transcription reaction (Thermoscript Reverse Transcriptase, Invitrogen, Karlsruhe, Germany) with a gene specific primer (GSP) binding to the *uidA *coding region in GT*Ds*B (R-GUS-D, 5'-CGCTGGCCTGCCCAACCTTT-3'). After RNA degradation with RNase H (Invitrogen, Karlsruhe, Germany) a homopolymeric tailing reaction with Terminal Deoxynucleotidyl Transferase (MBI Fermentas, St.Leon-Rot, Germany) and dGTP was carried out. The tailed cDNA was used as a template in a PCR with a tail binding Primer (CB3, 5'-CCCCCCCCTCCCCCCC-3', H. Schmidt, unpublished) and a nested GSP (R-GUS-B, 5'-CGCGCTTTCCCACCAACGCT-3'). 1 μl PCR product was subjected to a second PCR with CB3 and a third GSP (R-GUS-A, 5'-CCCACAGGCCGTCGAGTTT-3'). Specific DNA fragments were extracted from agarose gels and subcloned for analysis.

## Authors' contributions

KL designed the study, performed the experimental work and wrote the manuscript. SL provided GT*Ds*B and TPase barley lines, participated in the analysis of GT*Ds*B flanking sequences, in the design and coordination of the study and in the writing of the manuscript. All authors read and approved the final manuscript.
